# Spontaneous Septic Arthritis of Pubic Symphysis in an Elite Athlete

**DOI:** 10.1155/2016/6384707

**Published:** 2016-09-14

**Authors:** F. Jasmijn Smits, Herman Frima, Christoph Schaeffeler, Christoph Sommer

**Affiliations:** ^1^Department of Surgery, Kantonsspital Graubünden, Loëstrasse 170, 7000 Chur, Switzerland; ^2^Department of Surgery, University Medical Center Utrecht, Heidelberglaan 100, 3584 CX Utrecht, Netherlands; ^3^Department of Radiology, Kantonsspital Graubünden, Loëstrasse 170, 7000 Chur, Switzerland

## Abstract

Septic arthritis of the pubic symphysis is a potentially severe disease. Athletes are at risk of this form of spontaneous arthritis, as inflammation of the pubic bone due to muscular stress is relatively common. Oedema due to inflammation might predispose to infection through bacteraemia or local bacterial translocation. Suspicion should be raised when an athlete complains of groin pain and has signs of infection (i.e., fever, elevated white blood cell count, and elevated C-reactive protein). Diagnosis is made by imaging showing signs of inflammation combined with positive (blood) cultures. Broad spectrum antibiotics should be started upon suspicion and adjusted according to cultures. An abscess causing clinical deterioration under antibiotic treatment is an indication for invasive intervention (i.e., surgical or image-guided drainage). This is the first case of spontaneous septic arthritis of the pubic symphysis in an athlete requiring surgical and additional image-guided drainage.

## 1. Introduction

Septic arthritis of the pubic symphysis is a rare, though potentially severe, condition. In athletes, the sterile inflammation of the pubic bone (i.e., osteitis pubis) is relatively common. Both conditions can have a similar presentation. Early distinction is essential, since there are important differences in treatment [1]. With this case report we aim to increase awareness of this condition in athletes, as we believe early and adequate treatment might improve outcome of these patients.

## 2. Case Report

**Figure 1:**
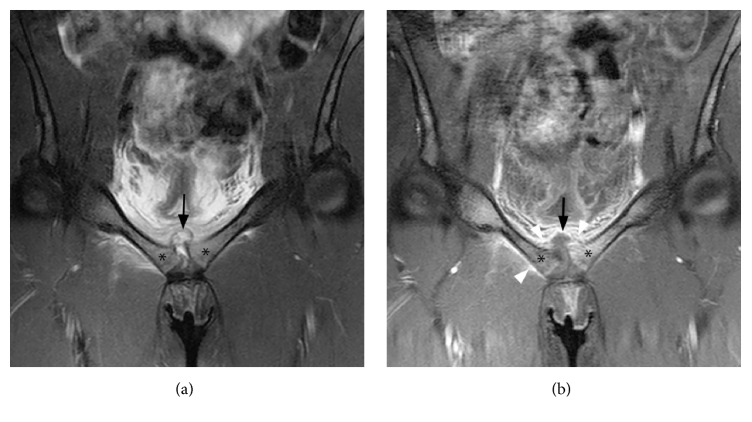
Corresponding coronal MR images of the pubic symphysis. (a) T2-weighted MR image with fat suppression showing bone marrow oedema in the pubic bones adjacent to the symphysis consistent with osteomyelitis (asterisk). A hyperintense fluid collection extending from the symphysis can be seen (black arrow). (b) T1-weighted MR image with fat suppression after intravenous Gadolinium administration showing marked contrast enhancement surrounding the hypointense fluid collection (black arrow) representing an abscess. There is also contrast enhancement representing inflammation in the surrounding soft tissues (white arrowheads) and within the pubic bones (asterisk).

A 16-year-old female semiprofessional climber presented with right hip and groin pain since 5 days. Initial complaints were referred to as musculoskeletal and treated symptomatically with nonsteroid anti-inflammatory drugs (NSAIDs). Her pain, however, increased excessively, causing total immobilisation overnight. On arrival in our emergency department, she had inguinal pain at right hip motion and compression pain over the pubic bone. She was feverish with a normal white blood cell count (WBC) and an elevated C-reactive protein (CRP) of 118 mg/l. After blood cultures were taken, she was admitted for intravenous antibiotic treatment (Amoxicillin/clavulanic acid, 3 doses of 2000/200 mg). Magnetic resonance (MR) imaging of the pelvis showed massive oedema of parasymphyseal pubic bone and soft tissues and retrosymphyseal abscess, suggestive of arthritis of the pubic symphysis (Figure 1). Blood cultures, taken on admission, were positive for* Staphylococcus aureus*, and antibiotic treatment was switched to Flucloxacillin, 4 doses of 2000 mg.

On the third day of admission, she remained feverish and immobile due to hip pain and her CRP rose to 150 mg/l under intravenous antibiotic treatment. Ultrasound showed polyserositis with pleural effusion, pericardial fluid, and ascites and the known abscess. Cardiac sonography revealed no signs of pericarditis. Because of clinical deterioration under antibiotic treatment, surgical intervention was required. The abscess was surgically explored and drained through a Pfannenstiel incision. Following that a diagnostic laparoscopy showed yellow but clear ascites without any abdominal infection.

After initial improvement, she developed tachypnea and saturation dropped as far as to 91% on the third day after surgical drainage. Computed tomography of the thorax, made to rule out pulmonary embolism, showed pneumonia. Antibiotic treatment was switched to Piperacillin, 3 doses of 4500 mg, and Clindamycin, 3 doses of 900 mg. MR imaging eight days after admission showed recurrence of the retrosymphyseal abscess. A percutaneous drain was placed in this abscess under ultrasound guidance.

**Figure 2:**
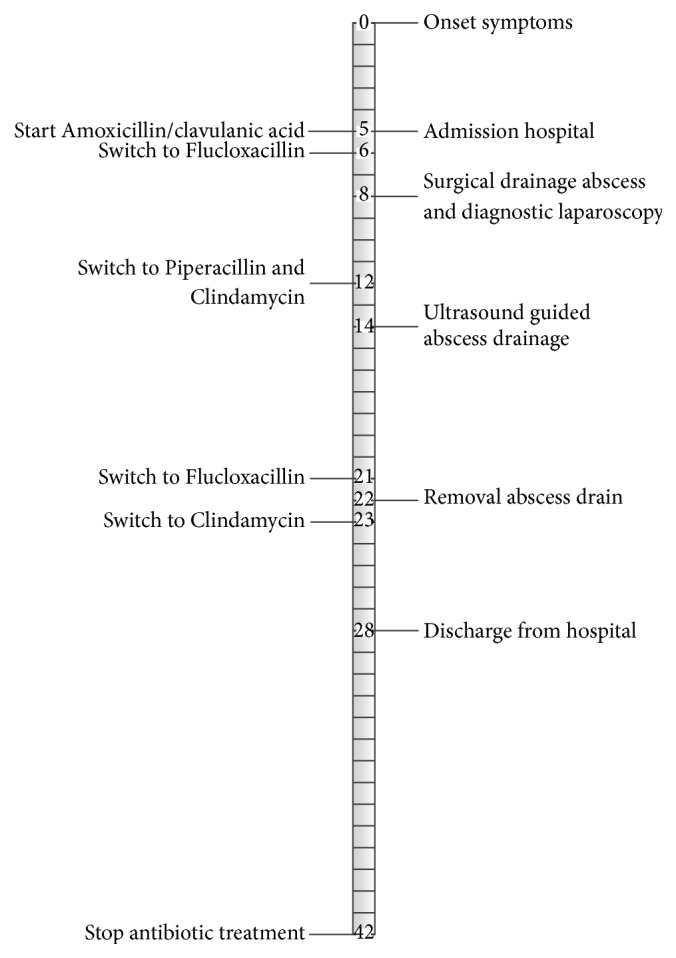
Timeline on clinical course; numbers indicate days after onset of symptoms.

Hereafter the patient showed clinical improvement. Antibiotic treatment was switched back to Flucloxacillin. She started mobilisation 13 days after admission. After 22 days in the hospital she could be discharged. Treatment through Clindamycin was continued for three more weeks. At this time total length of antibiotic treatment was six weeks (Figure 2, timeline). Two months after initial diagnosis she was fully mobile and back to school. Five months after diagnosis she started climbing again. She had only little complaints of groin start-up pain and a tight feeling in the adductor region. She showed no signs of infection after discharge.

## 3. Discussion

We present a case of a young athlete with spontaneous bacterial arthritis of the pubic symphysis. This is the only reported case of spontaneous septic symphysis in a female athlete requiring surgical intervention.

There is no current literature on female athletes or on patients with severe septic arthritis of the pubic symphysis (i.e., requiring surgical intervention). To date, a total of 10 athletes with proven septic arthritis of the pubic symphysis have been described [1–3]. All patients were male, aged 16 to 48 years. Amongst them were football players (*n* = 4), runners (*n* = 2), a fitness trainer, a soccer player, a weight lifter, and a soldier. All patients presented with groin pain during motion and fever. MR imaging of the pelvis, performed in 7/10 patients, showed oedema of pubic symphysis and surrounding structures. In one patient a small retrosymphyseal abscess was seen, which was deemed undrainable due to its size and position. Pathogens in blood cultures or aspirates were* Staphylococcus aureus* (*n* = 8),* Fusobacterium necrophorum* (*n* = 1), or* Staphylococcus epidermidis* (*n* = 1). All previously reported athletes were treated successfully with antibiotics; length of treatment was 6 to 8 weeks.

Osteitis pubis is a sterile inflammation of the pubic bone. This condition, caused by muscular stress on the pubic bone, is not uncommon in athletes [4]. Clinical presentation of osteitis pubis and septic arthritis of the pubic symphysis can be similar, with groin pain on motion and signs of inflammation. However, septic arthritis usually has an acute onset and is accompanied by fever, as opposed to osteitis [5]. When septic arthritis of the pubic symphysis is suspected, blood cultures should be taken before starting intravenous antibiotic treatment. The most common pathogen in athletes is* Staphylococcus aureus* [6]. Imaging of the pelvis, ideally through MR imaging, might show soft tissue inflammation and bone marrow oedema [7]. Primary treatment of osteitis pubis includes NSAIDs and corticosteroids, as treatment of osteomyelitis is primarily through antibiotics [1]. The presence of a retrosymphyseal abscess combined with lack of clinical improvement under antibiotic treatment is an indication for invasive treatment (i.e., surgical or image-guided drainage) [1].

Although differentiation between inflammatory (osteitis pubis) and infective condition (osteomyelitis pubis including arthritis of the pubic symphysis) is important to determine adequate treatment, there might also be a causative relation. Osteitis pubis is an inflammatory condition of the pubic bone that causes surrounding oedema. This might be a predisposing factor for infection following bacteraemia or translocation. Hypothetically osteitis and osteomyelitis are not two different entities but a continuum on the same spectrum [8].

Our case is the first describing severe spontaneous bacterial arthritis of the pubic symphysis in an athlete requiring surgical and image-guided drainage. Diagnosing septic arthritis of the pubic symphysis has been difficult in many of the published cases. This might be due to two major reasons. First, there is only little awareness as this is a rare condition. Second, the clinical presentation is similar to more frequent inflammation of the pubic bone (i.e., osteitis pubis). This case shows septic arthritis of the pubic symphysis can be the cause of sepsis and possibly need for surgical drainage.

## 4. Conclusion

We report a case of severe septic arthritis of the pubic symphysis in a female elite athlete. As this is a potentially severe condition, clinicians should be aware of this condition in feverish athletes with acute groin pain.
